# What is the Functional Role of Obturator Externus Muscle? The Case of a Moderate Tear in a Professional Soccer Player: A Case Report

**DOI:** 10.5704/MOJ.2603.021

**Published:** 2026-03

**Authors:** M Abate, G Cocco, L Sammarchi, V Salini

**Affiliations:** 1 Department of Orthopaedics, IRCCS Ospedale San Raffaele, Milano, Italy; 2 Institute of Advanced Biomedical Technologies, Università degli Studi Gabriele d'Annunzio, Chieti, Italy; 3 Department of Radiology, Habilita S.p.A Institute, Bergamo, Italy

**Keywords:** obturator externus muscle, soccer, Musculo-skeletal imaging, muscular strength, return to play

## Abstract

The obturator externus muscle is a pelvic and hip muscle with a complex anatomical arrangement, which is involved in hip stability. This paper presents a new case of an obturator externus muscle tear in a professional soccer player, offering valuable insights into the clinical presentation, diagnostic challenges, and successful conservative management. Additionally, a comprehensive review of the literature on this topic is presented. A 21-year-old player reported anterior hip and adductor discomfort after a game, with no recollection of a specific traumatic event. Clinical examination revealed tenderness and limited range of motion during external hip rotation with flexion. Ultrasonography was inconclusive, but magnetic resonance imaging showed a partial obturator externus muscle tear. The athlete was submitted to a structured exercise program and, despite the injury, continued training and playing without functional limitations. The magnetic resonance imaging, performed after 35 days, revealed a complete recovery. This paper highlights the rarity of obturator externus muscle tears, their potential underreporting, and the diagnostic value of magnetic resonance imaging in identifying these deep-seated injuries. Importantly, the player's rapid recovery and ability to continue playing suggest that conservative management can lead to excellent functional outcomes. The possible reasons explaining the complete recovery, including the role of this muscle in hip stability and individual anatomical variations, are discussed.

## Introduction

The obturator externus (OE) muscle, situated in the pelvic and hip region, together with the others deep external rotators muscles known as “short lateral rotators”, contributes to the active stability of the hip joint from tension and acts as postural muscle^1^.

Indirect injuries to the OE are relatively rare but can be sometimes implicated in the onset of acute anterior hip or groin pain in athletes. Because the clinical diagnosis can be challenging, radiological assessments (Magnetic Resonance Imaging [MRI] as gold standard) become necessary. The optimal management of these injuries is not well defined, especially in athletes, with return to play (RTP) typically ranging from 0 to 23 days^2^.

The purpose of this case report is to describe a new case of an acute moderate OE tear that occurred in a professional soccer player who was closely monitored with MRI imaging and clinical follow-up.

## Case Report

The procedure followed were in accordance with the Declaration of Helsinki and informed written consent was obtained from the subject described in this report. Internal review board was not necessary for the nature of the study.

**Fig. 1:**
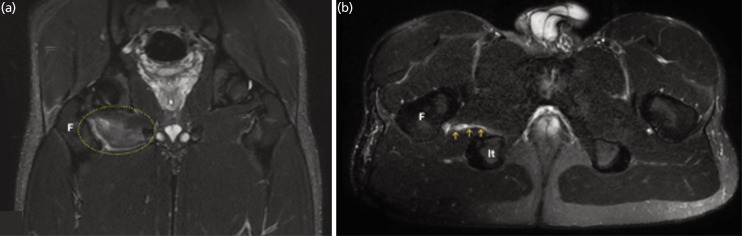
MRI images of moderate indirect right OE tear (STIR and T2 fat sat images). After 2 days from the event, in panel A (coronal, STIR) and B (axial, T2 fat), a moderate lesion of OE muscle can be observed (yellow circle). (a) In particular, a hyper-intense area can be appreciated into the muscular belly. (b) At the site of lesion, a peri-fascial fluid collection (hyper-intense area, yellow arrows), attributable to a sero-hematoma, is also present. (F: femur; It: ischial tuberosity).

A 21-year-old (BMI: 22,06kg/m^2^) professional soccer player (African origin, right-sided midfielder, Italian Second Division) reported blurred discomfort in his right anterior hip and adductor muscle areas (dominant leg) the day after an official game. He did not recall any traumatic event prior to the day he began experiencing discomfort. Because the clinical observation and ultrasound examination were inconclusive, a 1.5 T MRI was performed the following day. In the axial, sagittal, and coronal scans, a partial disruption of muscle fibres in the OE muscle belly with peri-fascial extension, associated with a fluid collection and edema, was observed (Fig. 1). These findings were suggestive of an isolated indirect moderate lesion (3A according to Munich classification). Additionally, degenerative alterations were observed in the pubic symphysis (i.e. sub-chondral sclerosis, bone edema, erosions, and cortical irregularities). The hip structures appeared normal with no signs of effusion, bony, chondral, or labral pathologies, and there was no damage to the vascular-nervous local system.

**Fig. 2:**
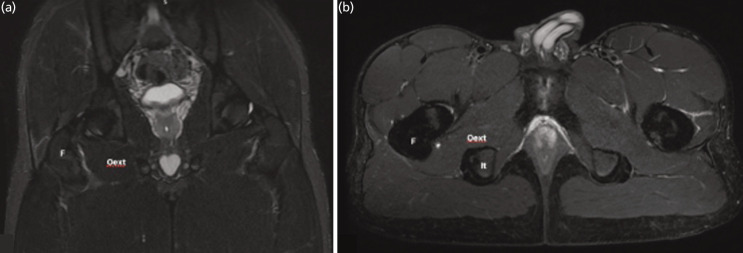
Control MRI performed after 35 days (STIR and PD fat sat images). In panel A (coronal, STIR) and B (axial, PD fat sat), MRI images demonstrate complete healing of OE. No area consistent with muscular tear can be observed. (F: femur; It: ischial tuberosity; Oext: obturator externus).

Considering the clinical presentation, radiological findings, and the player's minimal discomfort with no significant functional limitation, the medical staff allowed the athlete to continue training with the team. However, he followed a structured and supervised exercise program. He participated in an official game the week after the event. During the following weeks the player did not miss any training day, did not show any impact on his physical performance and remained at disposal for official games. After 35 days, a 1.5 T MRI control showed complete recovery of the OE muscle (Fig. 2).

## Discussion

This report presents a new case of an isolated tear in the OE muscle that occurred in a professional soccer player. Such lesions are exceptionally rare; from a comprehensive review of the literature, a total of thirty-one cases of OE strain have been reported in athletes (Table I)^2,3^.

**Table I:** Obturator externus injuries reported in literature.

Author (year)	Study design	N. of cases	Age	Sport activity	MRI grading	Associated lesions	Injury mechanism	RTP
Silva R, Pereira A, Rodrigues-Gomes S, Lopes T. An Unlikely Cause of Groin Pain: Obturator Externus Tear in a Professional Soccer Player. *Cureus.* 2023; 15(9): e44612. doi: 10.7759/cureus.44612	Case report	1	32	Soccer	Moderate	No	Indirect	23 days
Rhim HC, Gureck AE, Jang KM. Acute Obturator Externus Injury in Professional Soccer Players: A Case Series. *Medicina (Kaunas)*. 2022; 58(9): 1145. doi: 10.3390/medicina58091145	Case series	3	24, 24, 30	Soccer	Moderate	No	Indirect	10 days
Serner A, Weir A, Tol JL, Thorborg K, Roemer F, Guermazi A, *et al*. Characteristics of acute groin injuries in the adductor muscles: A detailed MRI study in athletes. *Seand J Med Sci Sports*. 2018; 28(2): 667-76. doi: 10.1111/sms. 12936	Prospective	9	N.A.	Different sports	Moderate/high	Yes	Indirect	N.A.
Girdwood M, West L, Connell D, Brukner P. Contact- Related Strain of Quadratus Femoris, Obturator Externus, and Inferior Gemellus in an Australian Football Player: A Case Report. *J Sport Rehabil*. 2019; 28(8): 887- 90. doi: 10.1123/jsr.2018-0279	Case report	1		American football	Moderate	Yes	Contact	8 weeks
Wong-On M, Turmo-Garuz A, Arriaza R, Gonzalez de Suso JM, Til-Perez L, Yanguas-Leite X, et al. Injuries of the obturator muscles in professional soccer players. *Knee Surg Sports Traumatol Arthrosc*. 2018; 26(7): 1936- 42. doi: 10.1007/s00167-017-4453-6	Retrospective observational	12	18-36	Soccer	Moderate/high	No	Indirect	11.5 ± 8.8 days ^
Khodaee M, Jones D, Spittler J. Obturator Internus and Obturator Externus Strain in a High School Quarterback. *Asian J Sports Med.* 2015; 6(3): e23481. doi: 10.5812/asjsm. 23481	Case report	1	14	American football	High	Yes	Contact	6 weeks
Valente FIG, Marques FO, de Souza LDS, Abib TA, Ribeiro DC. Injury of the external obturator muscle in professional soccer athletes. *Rev Bras Med Esporte.* 2011; 17(1): 36-9.	Case series	4 *	N.A.	Soccer	Moderate	No	Indirect	13, 19, 20, 21 days

As in our case, patients cannot recall a specific traumatic event and, therefore, a high degree of clinical suspicion, as well as diagnostic imaging, is essential to differentiate this injury from other hip and groin pathologies. MRI provides an accurate evaluation of deep hip muscles and enables a firm diagnosis and a safe therapeutic approach.

Even the optimal treatment remains poorly defined, especially in athletic population, in nearly all observed cases, conservative therapies have yielded excellent functional outcomes^2,3^, with no direct relationship between the degree of muscular strain seen on imaging and RTP (Table I).

In our case, similarly to three athletes reported in the paper by Wang-On *et al*^2^, the player was allowed to resume sports activity immediately, without functional limitations or missed training days/games. It must be underlined that the demanding physical activity did not hinder the muscular recovery (observed with MRI images at 35 days after injury). Some hypotheses that may explain these findings can be proposed.

The first explanation may be related by the role of OE itself which is a joint and postural stabiliser (consisting of type I slow-twitch fibres) rather than a generating explosive force muscle (type II fast-twitch fibres).

Secondly, OE activity may be of limited relevance during specific hip movements, which are supported by the coordinated contractions of surrounding muscles (i.e. adductors, ilio-psoas, and rectus femoris muscles). This feature could be further improved by a structured exercise program like the one followed by our athlete, which can improve hip function.

In our opinion, steroid injections are not recommended for muscular injuries due to the harmful side effects and the potential doping implication. Platelet rich plasma may be considered in selected cases (e.g. severe muscle tears or tendon injuries) for its potential to promote healing.

Third, the function of the OE muscle may be influenced by individual and racial anatomical variations. For instance, it may be speculated that Africans individuals (as our player) show bigger frames, and so the possibility of having a bigger muscle and tendon size compared to Caucasian subjects^4^. Variations in joint structure, collagen profiles, muscle fibre composition, vascular efficiency, and bone mineral density may also influence strength and recovery times^5^.

The comprehensive clinical and diagnostic follow-up carried out, the structured and supervised exercise program followed and RTP described in this case, can be considered strength points of the present report. However, due to the nature of the study and the short follow-up period, these findings cannot be generalised.

From a practical standpoint, sports clinicians should be aware that discomfort in the hip region may also be linked to injuries of deep muscles. Therefore, when symptoms are unclear, an MRI evaluation should be considered. In the case of an OE tear, implementing a structured exercise protocol is recommended to optimise recovery and reduce the risk of re-injury. Based on clinical symptoms and performance, the player may also return to sport without restrictions.
